# Prospective observational study of atezolizumab plus nab-paclitaxel in patients with metastatic triple-negative breast cancer: JBCRG-C08/ATTRIBUTE

**DOI:** 10.1038/s41523-026-00997-y

**Published:** 2026-06-26

**Authors:** Tatsunori Shimoi, Nobuhiro Shibata, Masahiro Kitada, Michiko Tsuneizumi, Mitsugu Yamamoto, Yukinori Ozaki, Yumi Fujimoto, Sayuri Akiyoshi, Naoki Hashimoto, Miki Yamaguchi, Yutaka Yamamoto, Norikazu Masuda, Hiroshi Tada, Takashi Yamanaka, Yuichiro Kikawa, Tetsuhiko Taira, Shizuka Nakagawa, Taizo Takahashi, Mari S. Oba, Naoki Niikura

**Affiliations:** 1 Department of Medical Oncology, National Cancer Centre Hospital, Tokyo, Japan; 2 Department of Clinical Oncology, Kansai Medical University Hospital, Osaka, Japan; 3 Department of Breast Disease Centre, Asahikawa Medical University Hospital, Hokkaido, Japan; 4 Department of Breast Surgery, Shizuoka General Hospital, Shizuoka, Japan; 5 Department of Breast Oncology, National Hospital Organisation Hokkaido Cancer Centre, Hokkaido, Japan; 6 Department of Breast Medical Oncology, Cancer Institute Hospital of JFCR, Tokyo, Japan; 7 Division of Breast Oncology, Saitama Cancer Centre, Saitama, Japan; 8 Department of Breast Oncology, National Hospital Organisation Kyushu Cancer Centre, Fukuoka, Japan; 9 Department of Breast Surgery, Aomori Prefectural Central Hospital, Aomori, Japan; 10 Department of Breast Surgery, JCHO Kurume General Hospital, Fukuoka, Japan; 11 Department of Breast and Endocrine Surgery, Kumamoto University Hospital, Kumamoto, Japan; 12 Department of Breast Surgery, Graduate School of Medicine, Kyoto University, Kyoto, Japan; 13 Department of Surgery, Division of Breast and Endocrine Surgery, Tohoku University Hospital, Sendai, Miyagi Japan; 14 Department of Breast Surgery and Oncology, Kanagawa Cancer Centre, Yokohama, Kanagawa Japan; 15 Department of Breast Surgery, Kansai Medical University Hospital, Osaka, Japan; 16 Department of Medical Oncology, Social Medical Corporation Hakuaikai Sagara Hospital, Kagoshima, Japan; 17 Biometrics Department, Clinical Development Division, Chugai Pharmaceutical Co. Ltd., Tokyo, Japan; 18 Oncology Medical Science Department, Medical Affairs Division, Chugai Pharmaceutical Co. Ltd., Tokyo, Japan; 19 Department of Clinical Data Science, Clinical Research & Education Promotion Division, National Center of Neurology and Psychiatry, National Center of Neurology and Psychiatry Hospital, Tokyo, Japan; 20 Department of Breast Oncology, Tokai University School of Medicine, Isehara, Kanagawa Japan

**Keywords:** Cancer, Oncology

## Abstract

The JBCRG-C08/ATTRIBUTE (UMIN000041747) study assessed atezolizumab plus nab-paclitaxel’s safety and effectiveness in a metastatic triple-negative breast cancer population, including IMpassion130-ineligible patients. The primary outcome was incidence of investigator-assessed adverse events (AEs). Safety and full analysis populations included 149 and 148 patients, respectively; 4.0% of the full analysis population had Eastern Cooperative Oncology Group performance status ≥2 and 7.4% had a history/comorbidity of autoimmune disease (AID) at baseline. AEs occurred in 96.6% of the safety analysis population; serious AEs (SAEs), in 16.8%; and immune-related AEs (irAEs), in 30.9%. irAE incidence was 45.5% in patients with a history/comorbidity of AID. Notable SAEs included pneumonitis (3.4%), diarrhoea, pyrexia, adrenal insufficiency and cardiac failure (1.3% each). Two cases of Grade 5 pneumonitis occurred. Regarding effectiveness, median progression-free survival was 5.6 months (95% confidence interval, 4.2–6.7). This first analysis found no new safety concerns with atezolizumab combination therapy, although patients with AID require careful monitoring.

## Introduction

Triple-negative breast cancer (TNBC) accounts for ~11% of all breast cancers and is defined by the absence of hormone receptor (oestrogen receptor and progesterone receptor) expression, as well as a lack of overexpression or amplification of human epidermal growth factor receptor 2 (HER2)^1^. TNBC is an aggressive subtype and is associated with poorer outcomes, risk of recurrence and poor survival following distant metastasis (which are more likely to occur in the brain and lungs) compared with other breast cancer subtypes^1–7^. Despite recent progress, and while chemotherapy remains the backbone of systemic treatment, novel targeted therapies and immunotherapeutic approaches have expanded the therapeutic armamentarium for TNBC in recent years^8,9^.

Approximately one-third of patients diagnosed with stage II or III TNBC experience metastatic recurrence within 2 to 3 years, despite receiving standard-of-care chemotherapy^10^. Following metastasis, median survival ranges from 12 to 18 months, underscoring the critical need for effective therapeutic strategies beyond conventional chemotherapy^11^. In TNBC, programmed death-ligand 1 (PD-L1) is predominantly expressed on tumour-infiltrating immune cells (ICs), suppressing anticancer immune responses. Therefore, targeting the PD-L1 pathway represents a promising therapeutic approach in this subtype^12^.

Atezolizumab is an anti–PD-L1 antibody that restores anti-tumour T-cell activity. Atezolizumab plus nab-paclitaxel demonstrated significantly improved efficacy over placebo plus nab-paclitaxel and manageable safety, in PD-L1–positive metastatic TNBC (mTNBC) in the IMpassion130 trial^13,14^, leading to its approval and recommendation as a first-line standard therapy in international guidelines^15–17^. Atezolizumab plus nab-paclitaxel was approved in Japan in 2019 and has since been recommended as a standard therapy for PD-L1–positive TNBC by the Japanese Breast Cancer Society Clinical Practice Guidelines^18,19^. However, its clinical application in diverse real-world populations, including those with characteristics that met the exclusion criteria of IMpassion130, remains insufficiently characterised.

Despite the efficacy of atezolizumab plus nab-paclitaxel in clinical trials, real-world evidence remains limited. Moreover, immune-related adverse events (irAEs) are known to occur during treatment as well as after discontinuation; however, detailed characterisation of these events is limited. Given the aggressive nature, poor prognosis and limited therapeutic options associated with mTNBC, it is essential to evaluate the real-world effectiveness and safety of treatment regimens^20^.

To address this gap, the JBCRG-C08/ATTRIBUTE study (UMIN000041747) prospectively evaluated real-world safety and effectiveness in patients with PD-L1–positive mTNBC who were scheduled to initiate atezolizumab plus nab-paclitaxel. The study included those who would have been excluded from clinical trials, such as those with a history of autoimmune disease (AID), those receiving atezolizumab as a second- or third-line treatment or those with an Eastern Cooperative Oncology Group performance status (ECOG PS) ≥2.

## Results

A total of 151 patients were enrolled between 15 February 2021 and 26 April 2024 across 79 institutions in Japan. The safety analysis population included 149 patients, after excluding two patients who had not received atezolizumab. The full analysis population comprised 148 patients, after excluding one non-conforming case. The response-evaluable population consisted of 107 patients from those with measurable disease at baseline (per Response Evaluation Criteria in Solid Tumours [RECIST] version 1.1) (*n* = 123), after excluding those with inappropriate baseline imaging (*n* = 5) and those with ongoing treatment at the data cut-off (*n* = 11) (Supplementary Fig. 1). The median follow-up duration for the safety analysis population was 12.9 months.

### Patient and disease characteristics

The median age of patients in this real-world study was 59 years (Table 1). Most patients (96.0%) had an ECOG PS of 0 or 1. Eleven patients (7.4%) had a history or comorbidity of AID prior to study treatment initiation. Atezolizumab plus nab-paclitaxel was administered as first-line therapy in 70.5% of cases, second-line in 22.1% and third-line in 7.4%.

**Table 1:** Patient demographics, tumour characteristics and treatment history (safety analysis population)

Characteristic	*N* = 149
Median age, years (range)	59 (31–85)
Sex, *n* (%)	
Female	147 (98.7)
Male	2 (1.3)
Menopausal status, *n* (%)^a^	
Premenopausal	32 (21.8)
Postmenopausal	111 (75.5)
Unknown	4 (2.7)
ECOG PS, *n* (%)	
0	112 (75.2)
1	31 (20.8)
2	5 (3.4)
3	1 (0.7)
4	0
History or comorbidity of autoimmune disease, *n* (%)	11 (7.4)
Multiple sclerosis	1 (0.7)
Sjögren syndrome	1 (0.7)
Basedow disease	3 (2.0)
Hashimoto disease	3 (2.0)
Rheumatoid arthritis	1 (0.7)
Psoriasis	1 (0.7)
Paediatric rheumatism	1 (0.7)
Surgery of the primary tumour, *n* (%)	109 (73.2)
Site of metastatic disease, *n* (%)^b^	
Liver	31 (20.8)
Lung	74 (49.7)
Brain	8 (5.4)
Bone	39 (26.2)
Stage at initial diagnosis, *n* (%)	
Stage I–III	95 (63.8)
De novo Stage IV	48 (32.2)
Unknown	6 (4.0)
*BRCA1/2* gene mutation, *n* (%)	
Positive	19 (12.8)
Negative	77 (51.7)
Unknown or not measured	53 (35.6)
Treatment line, *n* (%)	
First-line	105 (70.5)
Second-line	33 (22.1)
Third-line	11 (7.4)
Prior systemic therapy, *n* (%)^c^	
Perioperative setting	62 (58.5)
Metastatic setting	17 (16.0)
Both perioperative and metastatic setting	27 (25.5)
Prior perioperative treatment regimen, *n* (%)	
Anthracyclines	81 (54.4)
Taxanes	79 (53.0)
Immune checkpoint inhibitors	2 (1.3)
Prior metastatic treatment regimen, *n* (%)	
Anthracyclines	7 (4.7)
Taxanes	12 (8.1)
Immune checkpoint inhibitors	1 (0.7)

*ECOG PS* Eastern Cooperative Oncology Group performance status.

^a^Male subjects were excluded from the total safety analysis population of 149 (*n* = 147).

^b^Multiple responses allowed.

^c^Forty-three patients without prior systemic treatment were excluded from the safety analysis population of 149 (*n* = 106).

### Safety

In the safety analysis population (*n* = 149), from treatment initiation to 30 days after treatment discontinuation, adverse events (AEs) were reported in 144 patients (96.6%; 95% confidence interval [CI], 92.3–98.9). The most common AEs were neutropenia in 72 patients (48.3%; 95% CI, 40.1–56.6), alopecia in 68 patients (45.6%; 95% CI, 37.5–54.0), and neuropathy peripheral in 66 patients (44.3%; 95% CI, 36.2–52.7). From 31 days to 6 months after treatment discontinuation, AEs were reported in four patients (2.7%; 95% CI, 0.7–6.7); these included adrenal insufficiency (*n* = 2), pneumonitis (*n* = 1), and palmar-plantar erythrodysesthesia syndrome (*n* = 1). Overall, from treatment initiation to 6 months after treatment discontinuation, AEs occurred in 144 patients (96.6%; 95% CI, 92.3–98.9). Serious (SAEs) occurring from treatment initiation to 30 days after treatment discontinuation were reported in 25 patients (16.8%; 95% CI, 11.2–23.8). Among these SAEs, 21 events were of National Cancer Institute Common Terminology Criteria for Adverse Events (CTCAE) severity Grade ≥3 (14.1%; 95% CI, 8.9–20.7) (Table 2). Two patients (1.3%; 95% CI, 0.2–4.8) experienced Grade 5 pneumonitis.

**Table 2:** SAEs occurring from study treatment initiation to 30 days after treatment discontinuation (safety analysis population)

SAEs^a^	Any grade, *n* (%, 95% CI)	Grade ≥3, *n* (%, 95% CI)^b^	Median onset, days (range)^c^
Any SAE	25 (16.8%; 11.2–23.8%)	21 (14.1%; 8.9–20.7%)	67 (1–499)
Gastrointestinal disorders			
Diarrhoea	2 (1.3%; 0.2–4.8%)	1 (0.7%; 0.0–3.7%)	26.5 (21–32)
Colitis	1 (0.7%; 0.0–3.7%)	0 (0%; 0.0–2.4%)	162 (162–162)
Gastritis	1 (0.7%; 0.0–3.7%)	1 (0.7%; 0.0–3.7%)	117 (117–117)
Nausea	1 (0.7%; 0.0–3.7%)	1 (0.7%; 0.0–3.7%)	117 (117–117)
Vomiting	1 (0.7%; 0.0–3.7%)	1 (0.7%; 0.0–3.7%)	100 (100–100)
Lower gastrointestinal haemorrhage	1 (0.7%; 0.0–3.7%)	1 (0.7%; 0.0–3.7%)	31 (31–31)
Respiratory, thoracic and mediastinal disorders			
Pneumonitis	5 (3.4%; 1.1–7.7%)	3 (2.0%; 0.4–5.8%)	81 (11–499)
Cough	1 (0.7%; 0.0–3.7%)	1 (0.7%; 0.0–3.7%)	67 (67–67)
Endocrine disorders			
Adrenal insufficiency	2 (1.3%; 0.2–4.8%)	2 (1.3%; 0.2–4.8%)	121 (85–157)
Hyperthyroidism	1 (0.7%; 0.0–3.7%)	1 (0.7%; 0.0–3.7%)	100 (100–100)
Hypopituitarism	1 (0.7%; 0.0–3.7%)	1 (0.7%; 0.0–3.7%)	117 (117–117)
Infections and infestations			
Cellulitis	1 (0.7%; 0.0–3.7%)	1 (0.7%; 0.0–3.7%)	150 (150–150)
Pneumonia	1 (0.7%; 0.0–3.7%)	1 (0.7%; 0.0–3.7%)	105 (105–105)
Device related infection	1 (0.7%; 0.0–3.7%)	1 (0.7%; 0.0–3.7%)	65 (65–65)
COVID-19	1 (0.7%; 0.0–3.7%)	1 (0.7%; 0.0–3.7%)	138 (138–138)
Metabolism and nutrition disorders			
Hyperglycaemia	1 (0.7%; 0.0–3.7%)	1 (0.7%; 0.0–3.7%)	183 (183–183)
Hyponatraemia	1 (0.7%; 0.0–3.7%)	1 (0.7%; 0.0–3.7%)	118 (118–118)
Decreased appetite	1 (0.7%; 0.0–3.7%)	1 (0.7%; 0.0–3.7%)	16 (16–16)
Blood and lymphatic system disorders			
Febrile neutropenia	1 (0.7%; 0.0–3.7%)	1 (0.7%; 0.0–3.7%)	15 (15–15)
Immune thrombocytopenia	1 (0.7%; 0.0–3.7%)	1 (0.7%; 0.0–3.7%)	432 (432–432)
Cardiac disorders			
Cardiac failure	2 (1.3%; 0.2–4.8%)	2 (1.3%; 0.2–4.8%)	246.5 (1–492)
General disorders and administration site conditions			
Pyrexia	2 (1.3%; 0.2–4.8%)	1 (0.7%; 0.0–3.7%)	11 (9–13)
Investigations			
Aspartate aminotransferase increased	1 (0.7%; 0.0–3.7%)	0 (0%; 0.0–2.4%)	43 (43–43)
Platelet count decreased	1 (0.7%; 0.0–3.7%)	1 (0.7%; 0.0–3.7%)	260 (260–260)
Injury, poisoning and procedural complications			
Infusion-related reaction	1 (0.7%; 0.0–3.7%)	1 (0.7%; 0.0–3.7%)	1 (1–1)
Neoplasms benign, malignant and unspecified (including cysts and polyps)			
Acute myeloid leukaemia	1 (0.7%; 0.0–3.7%)	1 (0.7%; 0.0–3.7%)	260 (260–260)
Nervous system disorders			
Seizure	1 (0.7%; 0.0–3.7%)	1 (0.7%; 0.0–3.7%)	4 (4–4)
Skin and subcutaneous tissue disorders			
Erythema multiforme	1 (0.7%; 0.0–3.7%)	1 (0.7%; 0.0–3.7%)	228 (228–228)

Of the safety analysis population, 120 patients (80.5%) completed observation up to 30 days after treatment discontinuation.

*AE* adverse event, *CI* confidence interval, *COVID-19* coronavirus disease 2019, *PT* preferred term, *SAE* serious adverse event, *SOC* System Organ Class.

^a^SAEs were assessed by the attending physician from the initiation of research treatment until 30 days after treatment discontinuation.

^b^Grade 5 events included two cases of pneumonitis.

^c^Time from start of treatment to onset of symptoms, median, days (range). The listing for AEs displays the SOC and PT.

The most common SAEs (reported in ≥2 patients) were pneumonitis in five patients (3.4%; 95% CI, 1.1–7.7), adrenal insufficiency in two (1.3%; 95% CI, 0.2–4.8), cardiac failure in two (1.3%; 95% CI, 0.2–4.8), diarrhoea in two (1.3%; 95% CI, 0.2–4.8) and pyrexia in two (1.3%; 95% CI, 0.2–4.8) (Table 2).

Between 31 days and 6 months after treatment discontinuation, one case (0.7%; 95% CI, 0.0–3.7) of palmar-plantar erythrodysesthesia syndrome was observed as an SAE. From treatment initiation to 6 months after treatment discontinuation, SAEs were observed in 26 patients (17.4%; 95% CI, 11.7–24.5) (Supplementary Table 1).

Discontinuation of atezolizumab due to AEs occurred in 21 patients (14.1%; 95% CI, 8.9–20.7) and discontinuation of nab-paclitaxel due to AEs occurred in 26 patients (17.4%; 95% CI, 11.7–24.5). For atezolizumab, 14 discontinuations were due to irAEs. These included pneumonitis (seven events), and one event each of alanine aminotransferase (ALT) increased, aspartate aminotransferase (AST) increased, colitis, cough, gastritis, pyrexia and immune thrombocytopenia.

irAEs were reported in 46 patients (30.9%; 95% CI, 23.6–39.0) from treatment initiation to 30 days after treatment discontinuation, in four patients (2.7%; 95% CI, 0.7–6.7) from 31 days to 6 months after treatment discontinuation and in 48 patients (32.2%; 95% CI, 24.8–40.4) from treatment initiation to 6 months after treatment discontinuation (Supplementary Table 1). The most common irAEs reported in >5 patients from treatment initiation to 30 days after treatment discontinuation were pneumonitis in 11 patients (7.4%; 95% CI, 3.7–12.8), hypothyroidism in nine patients (6.0%; 95% CI, 2.8–11.2) and adrenal insufficiency in eight patients (5.4%; 95% CI, 2.3–10.3). During the period from 31 days to 6 months after treatment discontinuation, the irAEs were adrenal insufficiency (*n* = 2; 95% CI, 0.2–4.8; one was Grade ≥3), pneumonitis (*n* = 1; 95% CI, 0.0–3.7) and palmar-plantar erythrodysesthesia syndrome (*n* = 1; 95% CI, 0.0–3.7; Grade ≥3).

**Fig. 1:**
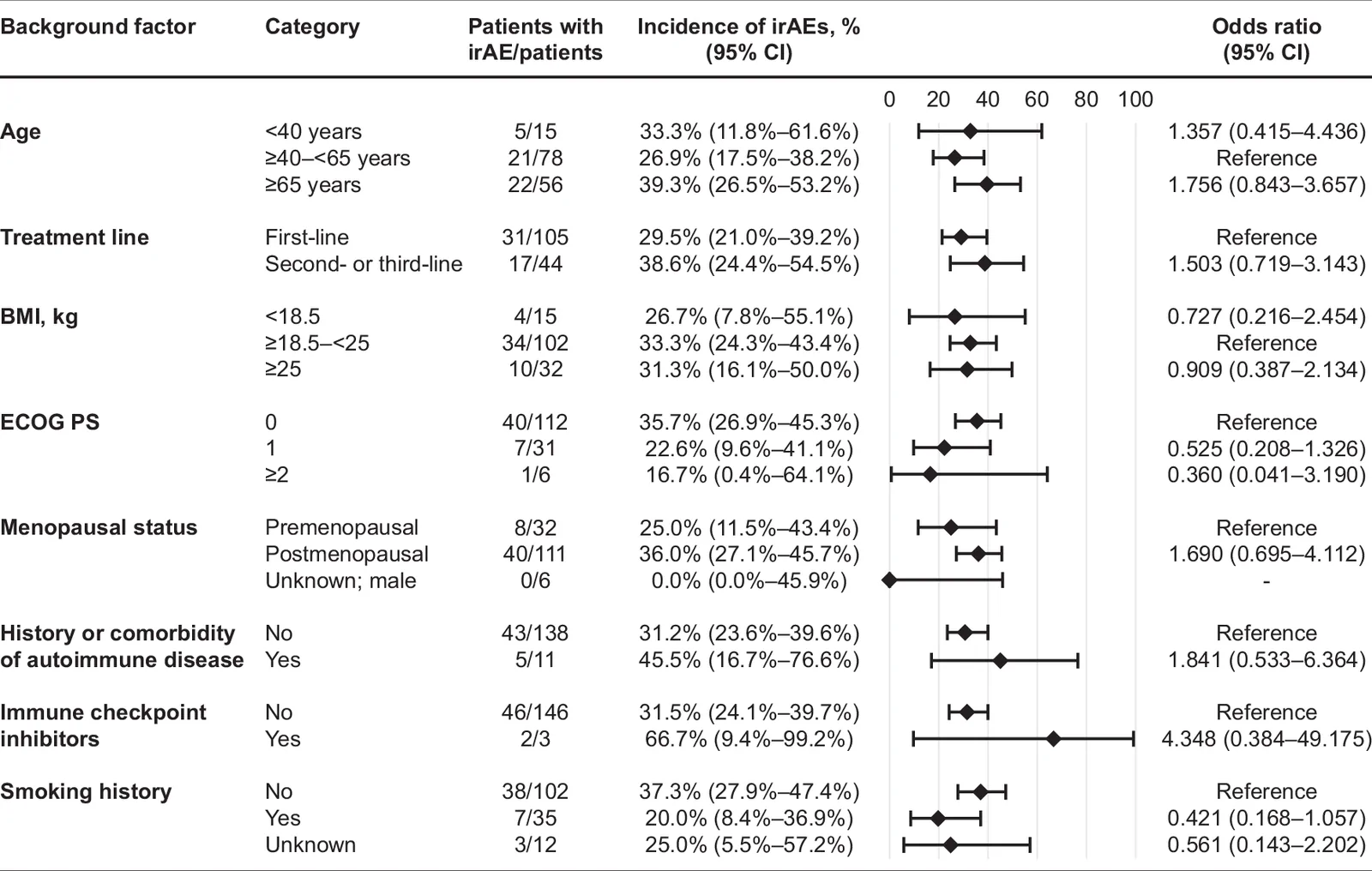
Incidence of irAEs by patient characteristics: analysis from treatment initiation to 6 months after treatment discontinuation (safety analysis population). BMI body mass index, CI confidence interval, ECOG PS Eastern Cooperative Oncology Group performance status, irAE immune-related adverse event.

**Fig. 2:**
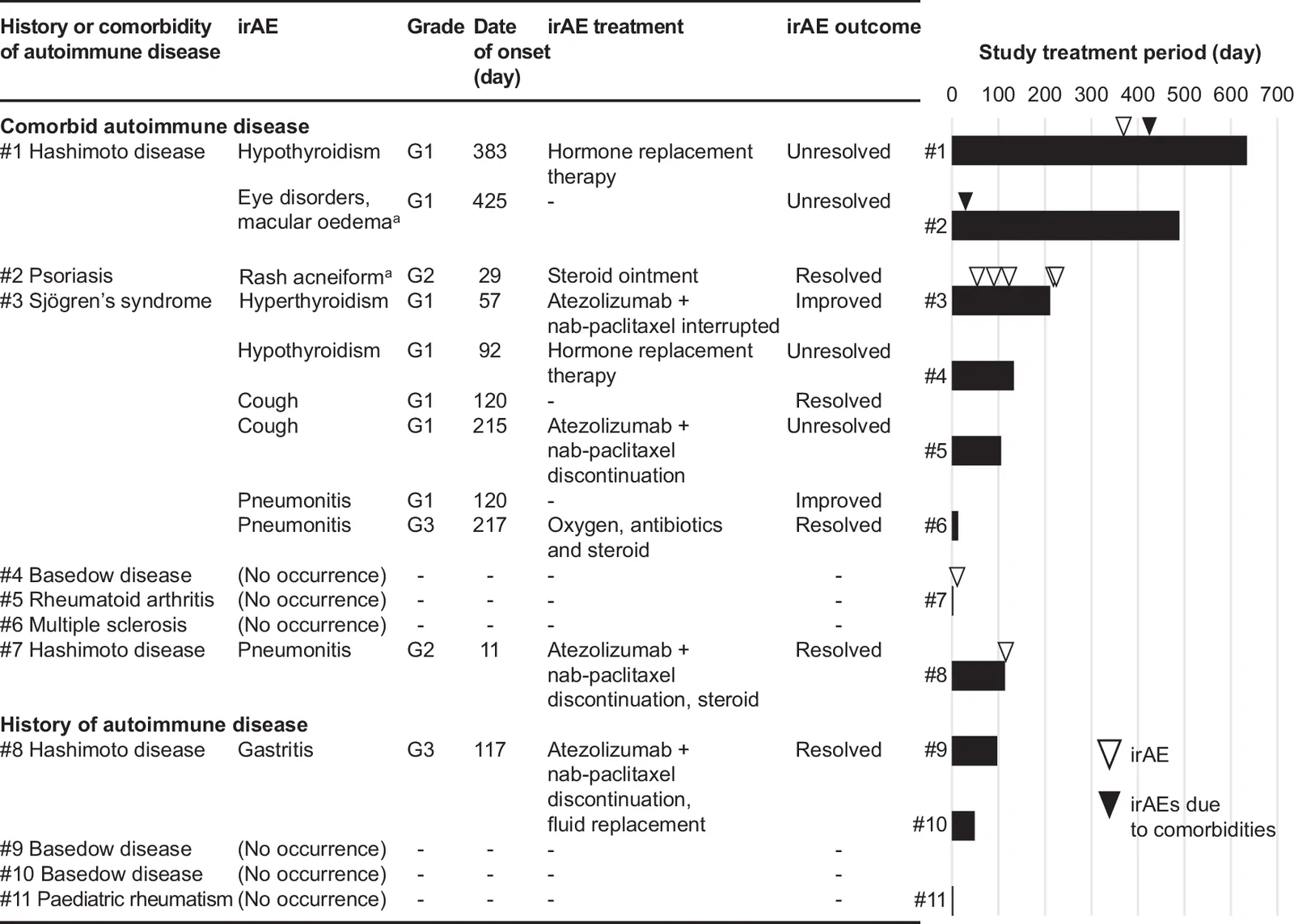
irAEs occurring from study treatment initiation to 6 months after treatment discontinuation (patients in the safety analysis population who have a history or comorbidity of autoimmune disease). *irAE* immune-related adverse event.

irAEs in the safety analysis population are shown in Fig. 1. irAE incidence in patients without vs. with a history or comorbidity of AID was 31.2% (95% CI, 23.6–39.6) vs. 45.5% (95% CI, 16.7–76.6); the odds ratio for irAEs in patients with AID compared to those without was 1.841 (95% CI, 0.533–6.364). Odds ratios with 95% CI for other subgroup comparisons are presented in Fig.1. The clinical courses and details of irAEs in the subgroup with a history or comorbidity of AID are shown in Fig. 2. This subgroup included 11 patients with a history or comorbidity of AID, and irAEs were observed in five of these patients. Two Grade 3 irAEs occurred, which were pneumonitis (*n* = 1) and gastritis (*n* = 1). Notably, two of the 11 patients who experienced irAEs had events considered to be exacerbations of their underlying AID: rash acneiform in a patient with psoriasis and hypothyroidism in a patient with Hashimoto disease.

irAE incidence in first-line vs. second-/third-line atezolizumab treatment was 29.5% (95% CI, 21.0–39.2) vs. 38.6% (95% CI, 24.4–54.5). irAE incidence in ECOG PS 0 vs. ECOG PS 1 vs. ECOG PS ≥2 was 35.7% (95% CI, 26.9–45.3) vs. 22.6% (95% CI, 9.6–41.1) vs. 16.7% (95% CI, 0.4–64.1).

### Effectiveness

Objective response rate (ORR) in the response-evaluable population was 36.4% (95% CI, 27.4–46.3), with seven patients (6.5%; 95% CI, 2.7–13.0) achieving complete response and 32 patients (29.9%; 95% CI, 21.4–39.5) achieving partial response. Twenty-five patients (23.4%; 95% CI, 15.7–32.5) had stable disease, including five patients (4.7%; 95% CI, 1.5–10.6) with stable disease lasting ≥24 weeks. Thirty-nine patients (36.4%; 95% CI, 27.4–46.3) had progressive disease and four patients (3.7%) were not evaluable. Clinical benefit rate (CBR) was 41.1% (95% CI, 31.7–51.0).

In patients who received first-line treatment and had measurable disease at baseline (*n* = 78), the ORR was 43.6% (95% CI, 32.4–55.3) and the CBR was 47.4% (95% CI, 36.0–59.1).

**Fig. 3:**
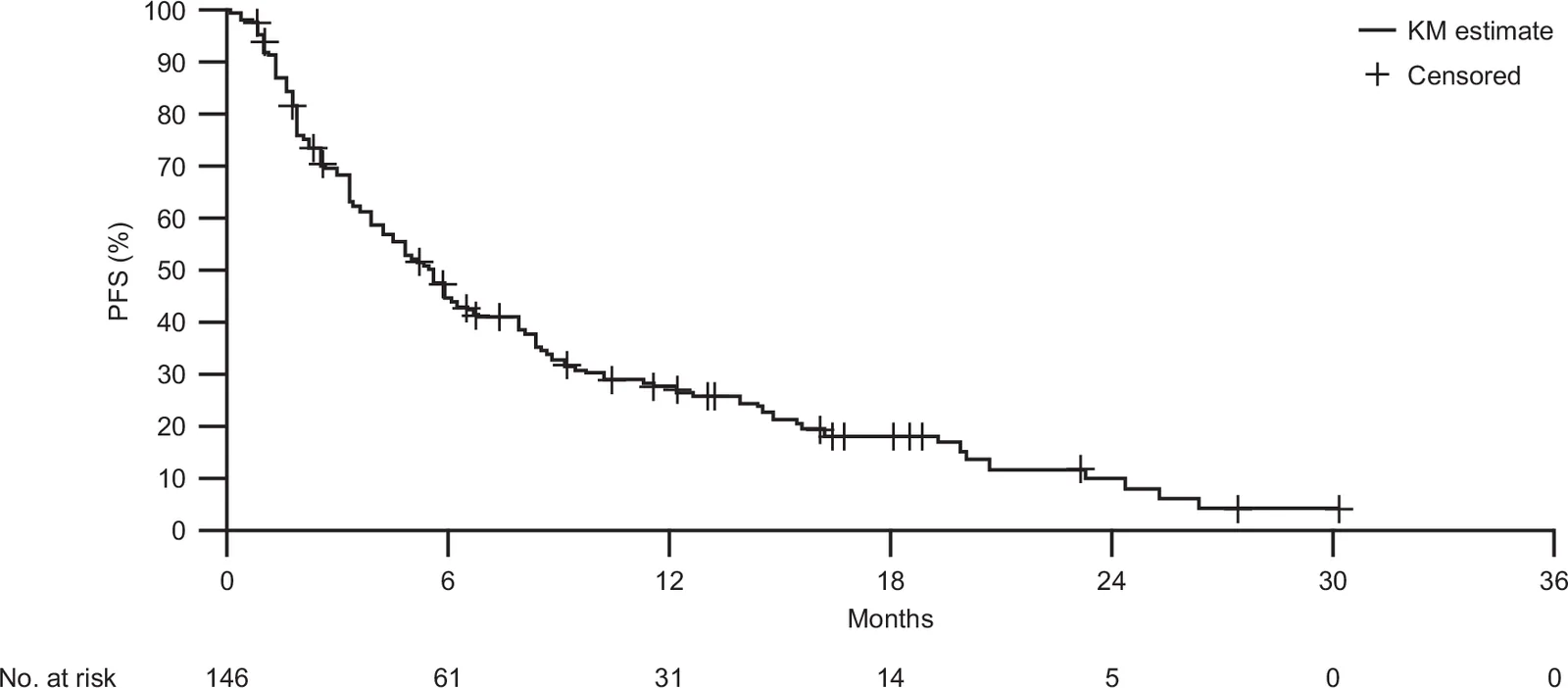
PFS (full analysis population). KM Kaplan–Meier, PFS progression-free survival. Two patients were excluded because PFS could not be calculated.

The median progression-free survival (PFS) in the full analysis population was 5.6 months (95% CI, 4.2–6.7) (Fig. 3). The median PFS was 5.7 months for patients who received atezolizumab as first-line treatment and 4.6 months for those who had second-/third-line treatment (hazard ratio; 1.21, 95% CI, 0.82–1.79) (Supplementary Table 2).

The median overall survival (OS) was 22.5 months (95% CI, 17.3–26.1) (Supplementary Fig. 2).

## Discussion

This multi-institutional, prospective, observational study describes the characteristics and outcomes for 149 patients treated with atezolizumab plus nab-paclitaxel in real-world practice. The study included patients who would have been excluded from enrolment in the IMpassion130 trial, such as patients with ECOG PS ≥2, those who previously received 1–2 lines of systemic treatment for mTNBC and those with a history or comorbidity of AID^21^.

The findings of the study showed a lower SAEs rate compared with the IMpassion130 trial (16.8% vs. 23.9%)^14^. This discrepancy likely reflects the fundamental differences in safety monitoring and reporting methodologies between a structured clinical trial and routine clinical practice. Clinical trials mandate intensive monitoring and structured AEs capture, which tends to ensure comprehensive data collection. In contrast, it is a well-established limitation that observational studies are susceptible to under-ascertainment and underreporting of AEs. Indeed, a systematic review on this topic has shown that AEs tend to be underreported in spontaneous reporting in real-world clinical practice^22^. Therefore, the lower SAE rate observed in our study may reflect the inherent limitations of data capture in a real-world setting. While a prospective observational study may reduce the likelihood of missing SAEs due to the severity of such events, the possibility of underreporting cannot be entirely excluded. The differences in incidence rates can be attributed to the broader data collection period in the IMpassion130 trial, which included SAEs reported up to 30 days after treatment as well as those occurring beyond 31 days^14^. Previous data also included patients with a negative PD-L1 status^14^.

The effectiveness outcomes observed in our real-world analysis were numerically lower than those reported in the IMpassion130 clinical trial^14^. This discrepancy likely reflects inclusion of patients with ECOG PS ≥2, prior lines of therapy, or autoimmune comorbidities, who are systematically excluded from pivotal trials but commonly encountered in practice. These results illustrate the challenge of extrapolating trial efficacy to effectiveness in heterogeneous real-world populations.

No new safety signals were observed in this real-world analysis. The study showed that irAEs can occur for up to 6 months after treatment discontinuation, extending beyond conventional trial observation periods. This real-world evidence aligns with Society for Immunotherapy of Cancer recommendations^23^ for long-term monitoring and underscores the need for structured survivorship care models that address delayed immune-related toxicities.

The underlying mechanism of irAE occurrence is still largely unclear. The drug elimination half-life is considered one of the mechanisms for post-treatment irAE occurrence. The elimination half-life of atezolizumab is 27 days^24^, and it takes 5 months for complete elimination from the body after administration. Therefore, it is considered that irAEs may occur for extended periods after treatment discontinuation. Accordingly, some studies recommend continued monitoring every 3 months for the first year after immune checkpoint inhibitor (ICI) therapy, followed by biannual assessments thereafter^25,26^. Since irAEs can recur or appear with delayed onset after treatment discontinuation, continuous observation is necessary not only during treatment but also after treatment discontinuation.

Patients with AID experienced a higher incidence of irAEs (45.5%), consistent with previous reports^27,28^. Notably, most events were manageable, Grade 1–2 and did not lead to treatment discontinuation, indicating that cancer immunotherapy may be feasible in carefully selected patients through thorough risk–benefit assessment and multidisciplinary management. This finding broadens the potential treatment population beyond those typically included in clinical trials. These results indicate that while patients with a history or comorbidity of AID have a higher rate of irAEs, no new safety signals were identified. Evidence regarding the irAE profile of ICI therapy in patients with breast cancer and a history or comorbidity of AID is currently limited, and this study provides valuable insights into this clinically important population.

When administering ICIs to patients with a history or comorbidity of AID, treatment should be considered with careful assessment of risks and benefits^29^.

These findings align with the real-world ANASTASE study conducted in Italy, which evaluated atezolizumab plus nab-paclitaxel in PD-L1–positive mTNBC and reported safety and effectiveness outcomes consistent with the efficacy observed in the IMpassion130 trial, with no unexpected AEs^30^. Similarly, the global EL1SSAR study demonstrated comparable safety and efficacy profiles in a more selected population, further supporting the consistency of results with both this study and IMpassion130^31^.

Finally, while atezolizumab has been withdrawn for TNBC in some regions, our findings offer critical real-world insights for countries where atezolizumab remains available, and they underscore the importance of context-specific data to guide treatment decisions in a rapidly evolving therapeutic landscape.

This is an observational study conducted in a real-world setting; therefore, there were variations in visit timing, imaging evaluation intervals and the management of AEs. As an observational study, our analysis is subject to the inherent limitations of AE reporting in a real-world setting, including potential underreporting and under-ascertainment. AEs that are of lower grade or whose causal relationship to the study treatment is not immediately clear may be less likely to be documented. Consequently, the safety profile described in this study may not fully capture the complete incidence of all AEs.

Furthermore, the possibility of selection bias cannot be excluded, which may influence the generalisability of the findings. Given the median follow-up period of 12.9 months, many censored cases were included in the PFS and OS analyses, limiting the ability to draw definitive conclusions regarding long-term effectiveness.

Moreover, irAEs potentially associated with atezolizumab treatment were classified solely based on the clinical judgement of treating physicians, without validation through independent adjudication. Furthermore, in the case of late-onset AEs that influence subsequent treatments, it may be challenging to determine whether these irAEs were related to atezolizumab or whether results were confounded by other factors, including the effects of post-treatment therapies, as time has passed since treatment discontinuation, and the impact of subsequent treatments on outcomes has not been sufficiently evaluated.

This large-scale prospective study, which enrolled 151 patients with mTNBC, demonstrated the safety of atezolizumab plus nab-paclitaxel combination therapy in real-world clinical practice. Despite including patients who met the exclusion criteria of IMpassion130, atezolizumab plus nab-paclitaxel combination therapy had no new safety concerns, reinforcing its clinical utility in the real-world setting. However, treatment in patients with a history or comorbidity of AID resulted in a high incidence of irAEs. Treatment should be considered with careful assessment of risks and benefits.

## Methods

### Study design and patients

This was a multi-institutional, prospective, observational study. Eligible patients were aged 20 years or older, and had PD-L1–positive, hormone receptor-negative, HER2-negative, inoperable or recurrent breast cancer, with ≤2 prior systemic treatments for mTNBC (excluding endocrine therapy) and without a history of hypersensitivity to any component of atezolizumab, who were scheduled to receive atezolizumab plus nab-paclitaxel in clinical practice. PD-L1–positivity was determined using the VENTANA PD-L1 (SP142) assay (Roche Diagnostics K.K., Tokyo, Japan) and defined as PD-L1 expression on tumour-infiltrating ICs covering ≥1% of the tumour area (IC ≥1%). The planned end date of enrolment was 3 years and 10 months from the date of enrolment of the first study subject, or until the target sample size was reached, whichever occurred first. The end date of the observation period was 4 years and 10 months from the date of enrolment of the first study subject, or up to 2 years from the date of enrolment of the last study subject, whichever occurred first.

### Study treatments

The study treatment was defined as treatment with atezolizumab and nab-paclitaxel. However, if either atezolizumab or nab-paclitaxel was discontinued due to AEs, and the other treatment was continued, the study treatment was regarded as continued.

The recommended dosage and administration schedule consisted of atezolizumab 840 mg administered intravenously every 2 weeks, concurrently with nab-paclitaxel 100 mg/m² administered intravenously on Days 1, 8 and 15. However, alternative dosage and administration schedules were not considered protocol deviations. The study protocol recommended referring to the IMpassion130 trial for guidance on treatment interruption, discontinuation and dose reduction. However, specific criteria were not defined, and decisions were left to the investigator’s discretion. No post-treatment therapy was specified after study treatment discontinuation.

The study protocol recommended periodic imaging assessments for tumour evaluation. However, as this was an observational study without a specific imaging schedule, tumour assessments were conducted by investigators according to routine clinical practice. The end of study treatment was defined as the day of the final dose when both atezolizumab and nab-paclitaxel were terminated. If nab-paclitaxel alone was continued after the discontinuation of atezolizumab, and nab-paclitaxel with another drug was started, the end date of study treatment was the day of administration of nab-paclitaxel monotherapy immediately before the start of the combination therapy. If the study drug was not administered for 12 weeks or longer, the last dose at this time point was considered as the end date of study treatment.

### Outcomes

The primary outcome was incidence of investigator-assessed AEs. AE severity was graded according to CTCAE version 5.0. All AEs were collected from treatment initiation until 30 days after treatment discontinuation. The relationship between AEs and the study drug was determined by the investigator’s judgement. In this study, SAEs were defined according to the study protocol as AEs meeting any of the following criteria: 1) fatal; 2) life-threatening; 3) requiring inpatient hospitalisation or prolongation of existing hospitalisation; 4) resulting in persistent or significant disability/incapacity; 5) a congenital anomaly/birth defect; or 6) other medically important conditions requiring intervention to prevent one of the other serious outcomes. From 31 days to 6 months after treatment discontinuation, events judged as related to atezolizumab, which were categorised into two groups: 1) any SAEs, and 2) a predefined list of AEs of special interest, regardless of their seriousness, were collected. The list of AEs of special interest included: pneumonitis, Grade ≥3 hypoxia or dyspnoea, colitis, endocrine disorders (diabetes, pancreatitis or adrenal insufficiency), vasculitis, hepatitis, liver enzymes (AST >3 × upper limit of normal [ULN] and bilirubin >2 × ULN, ALT >3 × ULN and bilirubin >2 × ULN, AST >10 × ULN, ALT >10 × ULN), systemic lupus erythematosus, Guillain-Barré syndrome and skin reactions (vitiligo, bullous pemphigoid). irAEs were collected during treatment and after treatment discontinuation.

Secondary outcomes included PFS, ORR, CBR and OS. CBR was defined as the proportion of patients achieving complete response, partial response or durable stable disease (≥24 weeks). Effectiveness was assessed by investigators based on RECIST version 1.1.

### Statistical analysis and considerations

Approximately 150 patients were planned to be enrolled to ensure a reasonable chance of observing ≥1 rare AE (expected incidence 1.1%) for the primary objective of safety. The safety analysis population comprised patients who received ≥1 dose of atezolizumab following registration. The full analysis population for effectiveness analyses comprised the safety analysis population, excluding ineligible patients and those without effectiveness assessments.

For the primary analysis, the 95% CIs for the number and proportion of patients who experienced AEs were estimated. For time-to-event outcomes, survival functions were estimated using the Kaplan–Meier method, with survival rates at specific timepoints and 95% CIs calculated using Greenwood’s formula. For ORR and CBR, the numbers of eligible patients, as well as the numbers and proportions of responders with 95% CIs, were calculated. Confirmation of the duration of response according to RECIST was not necessary for this clinical study. Subgroup analyses were exploratory in nature and were not powered for formal statistical comparisons; therefore, no adjustment for multiplicity was performed.

All statistical analyses were performed using SAS version 9.4 (SAS Institute Inc., Cary, NC, USA). This report analysed data collected up to a cut-off set 6 months after completion of enrolment for all patients; effectiveness results should be regarded as preliminary and may change upon final analysis.

### Ethics

The study was done in conformance with Good Clinical Practice guidelines and the Declaration of Helsinki. The protocol was reviewed and approved by the institutional review board or ethics committee at each study site, including the National Cancer Center Hospital (approval number: 2020-442). All amendments affecting study recruitment and conduct were approved. All enrolled patients received a full explanation of the contents of this clinical research and provided written consent to participate.

## Supplementary information

Supplementary Information

## Data Availability

The datasets generated and/or analysed during the current study are not publicly available to protect patient privacy. However, the data can be made available from the corresponding author upon reasonable request.

## Code availability

No custom code was used in this study.
