# Supplementary material for: Prospective observational study of atezolizumab plus nab-paclitaxel in patients with metastatic triple-negative breast cancer: JBCRG-C08/ATTRIBUTE
Source: NPJ Breast Cancer. 2026 Jun 26;12:135. doi: 10.1038/s41523-026-00997-y (PMC13672298; doi:10.1038/s41523-026-00997-y)
Supplement: Supplementary file 1 [file 41523_2026_997_MOESM1_ESM.pdf]

Supplementary Tables

Supplementary Table 1. irAEs occurring from study treatment initiation to 6 months after treatment discontinuation (safety analysis population)

irAEs*	From treatment initiation to 30 days after treatment discontinuation	From 31 days to 6 months after treatment discontinuation	From treatment initiation to 6 months after treatment discontinuation
**Any AEs**	**46 (30.9%; 23.6–39.0%)**	**4 (2.7%; 0.7–6.7%)**	**48 (32.2%; 24.8–40.4%)**
**Endocrine disorders**			
Hypothyroidism	9 (6.0%; 2.8–11.2%)		9 (6.0%; 2.8–11.2%)
Adrenal insufficiency	8 (5.4%; 2.3–10.3%)	2 (1.3%; 0.2–4.8%)	9 (6.0%; 2.8–11.2%)
Hyperthyroidism	4 (2.7%; 0.7–6.7%)		4 (2.7%; 0.7–6.7%)
Hypopituitarism	2 (1.3%; 0.2–4.8%)		2 (1.3%; 0.2–4.8%)
**Respiratory, thoracic, and mediastinal disorders**			
Pneumonitis	11 (7.4%; 3.7–12.8%)	1 (0.7%; 0.0–3.7%)	12 (8.1%; 4.2–13.6%)
Cough	2 (1.3%; 0.2–4.8%)		2 (1.3%; 0.2–4.8%)
**Skin and subcutaneous tissue disorders**			
Eczema	2 (1.3%; 0.2–4.8%)		2 (1.3%; 0.2–4.8%)
Rash maculo-papular	2 (1.3%; 0.2–4.8%)		2 (1.3%; 0.2–4.8%)
Dermatitis acneiform	1 (0.7%; 0.0–3.7%)		1 (0.7%; 0.0–3.7%)
Erythema multiforme	1 (0.7%; 0.0–3.7%)		1 (0.7%; 0.0–3.7%)
Erythema nodosum	1 (0.7%; 0.0–3.7%)		1 (0.7%; 0.0–3.7%)
Skin disorder	1 (0.7%; 0.0–3.7%)		1 (0.7%; 0.0–3.7%)
Eosinophilic panniculitis	1 (0.7%; 0.0–3.7%)		1 (0.7%; 0.0–3.7%)
Superficial inflammatory dermatosis	1 (0.7%; 0.0–3.7%)		1 (0.7%; 0.0–3.7%)
Palmar-plantar erythrodysesthesia syndrome		1 (0.7%; 0.0–3.7%)	1 (0.7%; 0.0–3.7%)
**General disorders and administration site conditions**			
Fatigue	4 (2.7%; 0.7–6.7%)		4 (2.7%; 0.7–6.7%)
Pyrexia	4 (2.7%; 0.7–6.7%)		4 (2.7%; 0.7–6.7%)
Malaise	1 (0.7%; 0.0–3.7%)		1 (0.7%; 0.0–3.7%)
**Investigations**			
Alanine aminotransferase increased	4 (2.7%; 0.7–6.7%)		4 (2.7%; 0.7–6.7%)
Aspartate aminotransferase increased	4 (2.7%; 0.7–6.7%)		4 (2.7%; 0.7–6.7%)
Amylase increased	2 (1.3%; 0.2–4.8%)		2 (1.3%; 0.2–4.8%)
Lipase increased	1 (0.7%; 0.0–3.7%)		1 (0.7%; 0.0–3.7%)
Troponin I increased	1 (0.7%; 0.0–3.7%)		1 (0.7%; 0.0–3.7%)
Blood alkaline phosphatase increased	1 (0.7%; 0.0–3.7%)		1 (0.7%; 0.0–3.7%)
**Gastrointestinal disorders**			
Colitis	1 (0.7%; 0.0–3.7%)		1 (0.7%; 0.0–3.7%)
Constipation	1 (0.7%; 0.0–3.7%)		1 (0.7%; 0.0–3.7%)
Gastritis	1 (0.7%; 0.0–3.7%)		1 (0.7%; 0.0–3.7%)
Nausea	1 (0.7%; 0.0–3.7%)		1 (0.7%; 0.0–3.7%)
Vomiting	1 (0.7%; 0.0–3.7%)		1 (0.7%; 0.0–3.7%)
**Metabolism and nutrition disorders**			
Decreased appetite	2 (1.3%; 0.2–4.8%)		2 (1.3%; 0.2–4.8%)
Diabetes mellitus	1 (0.7%; 0.0–3.7%)		1 (0.7%; 0.0–3.7%)

**Blood and lymphatic system disorder**			
Anaemia	1 (0.7%; 0.0–3.7%)		1 (0.7%; 0.0–3.7%)
Neutropenia	1 (0.7%; 0.0–3.7%)		1 (0.7%; 0.0–3.7%)
Immune thrombocytopenia	1 (0.7%; 0.0–3.7%)		1 (0.7%; 0.0–3.7%)
**Eye disorders**			
Macular oedema	1 (0.7%; 0.0–3.7%)		1 (0.7%; 0.0–3.7%)
Conjunctival hyperaemia	1 (0.7%; 0.0–3.7%)		1 (0.7%; 0.0–3.7%)
**Musculoskeletal and connective tissue disorders**			
Arthralgia	1 (0.7%; 0.0–3.7%)		1 (0.7%; 0.0–3.7%)
**Nervous system disorders**			
Immune-mediated encephalitis	1 (0.7%; 0.0–3.7%)		1 (0.7%; 0.0–3.7%)

AE adverse event, *CI* confidence interval, *irAE* immune-related adverse event, *PT* preferred term, *SOC* System Organ Class.

irAEs assessed by the attending physician from the start of research treatment until 30 days after treatment discontinuation. irAEs causally related to atezolizumab (as determined by the attending physician) occurring between 31 days and 6 months after investigational drug discontinuation. The listing for AEs displaying the SOC and PT. Data: n (%; 95% CI). Of the safety analysis population, 120 patients (80.5%) completed observation up to 30 days after treatment discontinuation.

Supplementary Table 2. PFS in patient subgroups (full analysis population)

**Background factor**	**Category**	**Events/ patients, *n***	**Median PFS, months (95% CI)***	**Hazard ratio (95% CI)**
Age	<40 years	14/14	3.3 (1.9–6.6)	1.231 (0.692–2.190)
	≥40 to <65 years	69/77	4.6 (3.3–5.6)	Reference
	≥65 years	34/55	9.4 (6.2–12.7)	0.573 (0.379–0.868)
Treatment line	First-line	80/102	5.7 (4.2–7.9)	Reference
	Second- or third-line	37/44	4.6 (2.6–9.1)	1.212 (0.820–1.792)
BMI	<18.5 kg/m^2	10/15	5.0 (1.4–NA)	1.203 (0.619–2.336)
	≥18.5 to <25 kg/m^2	80/99	5.1 (3.5–7.9)	Reference
	≥25 kg/m^2	27/32	6.0 (3.9–9.8)	1.006 (0.647–1.566)
ECOG PS	0	89/111	5.6 (4.2–6.9)	Reference
	1	24/29	5.1 (3.3–8.5)	1.144 (0.728–1.798)
	≥2	4/6	2.3 (0.1–NA)	1.475 (0.540–4.030)
Menopausal status	Premenopausal	29/31	3.5 (2.2–5.0)	Reference
	Postmenopausal	83/109	6.9 (4.6–8.6)	0.674 (0.439–1.034)
	Unknown; male	5/6	5.4 (1.4–NA)	0.627 (0.239–1.645)
History or comorbidity of autoimmune disease	No	109/135	5.6 (4.0–7.9)	Reference
	Yes	8/11	4.9 (0.9–NA)	1.093 (0.532–2.247)
Liver metastasis	No	89/115	6.1 (4.9–8.3)	Reference
	Yes	28/31	3.3 (1.8–4.9)	1.715 (1.116–2.635)
Lung metastasis	No	56/74	6.0 (3.6–8.3)	Reference
	Yes	61/72	5.1 (3.5–6.2)	1.076 (0.747–1.549)
Brain metastasis	No	109/138	5.6 (4.3–7.9)	Reference
	Yes	8/8	2.4 (0.5–4.6)	2.734 (1.324–5.649)
PD-L1 status	Low (IC1)	78/92	4.9 (3.3–6.0)	Reference
	High (IC2 or more)	26/38	7.9 (4.9–16.2)	0.568 (0.362–0.890)
	Unknown	13/16	5.1 (1.9–14.4)	0.915 (0.508–1.647)
BRCA1/2 gene mutation	Positive	18/19	4.2 (2.0–7.9)	Reference
	Negative	59/75	6.1 (3.5–8.3)	0.764 (0.450–1.299)
	Unknown + including unmeasured	40/52	5.3 (3.3–8.3)	0.736 (0.421–1.287)
Smoking history	No	81/101	5.3 (3.7–8.3)	Reference
	Yes	28/33	5.0 (3.1–6.7)	1.200 (0.778–1.851)
	Unknown	8/12	6.6 (2.6–NA)	0.690 (0.333–1.430)

BMI body mass index, *CI* confidence interval, *ECOG PS* Eastern Cooperative Oncology Group performance status, *IC* immune cell, *NA* not applicable, *PFS* progression-free survival.

* Two cases were excluded due to the inability to calculate PFS.

Supplementary Fig. 1. Patient flow diagram

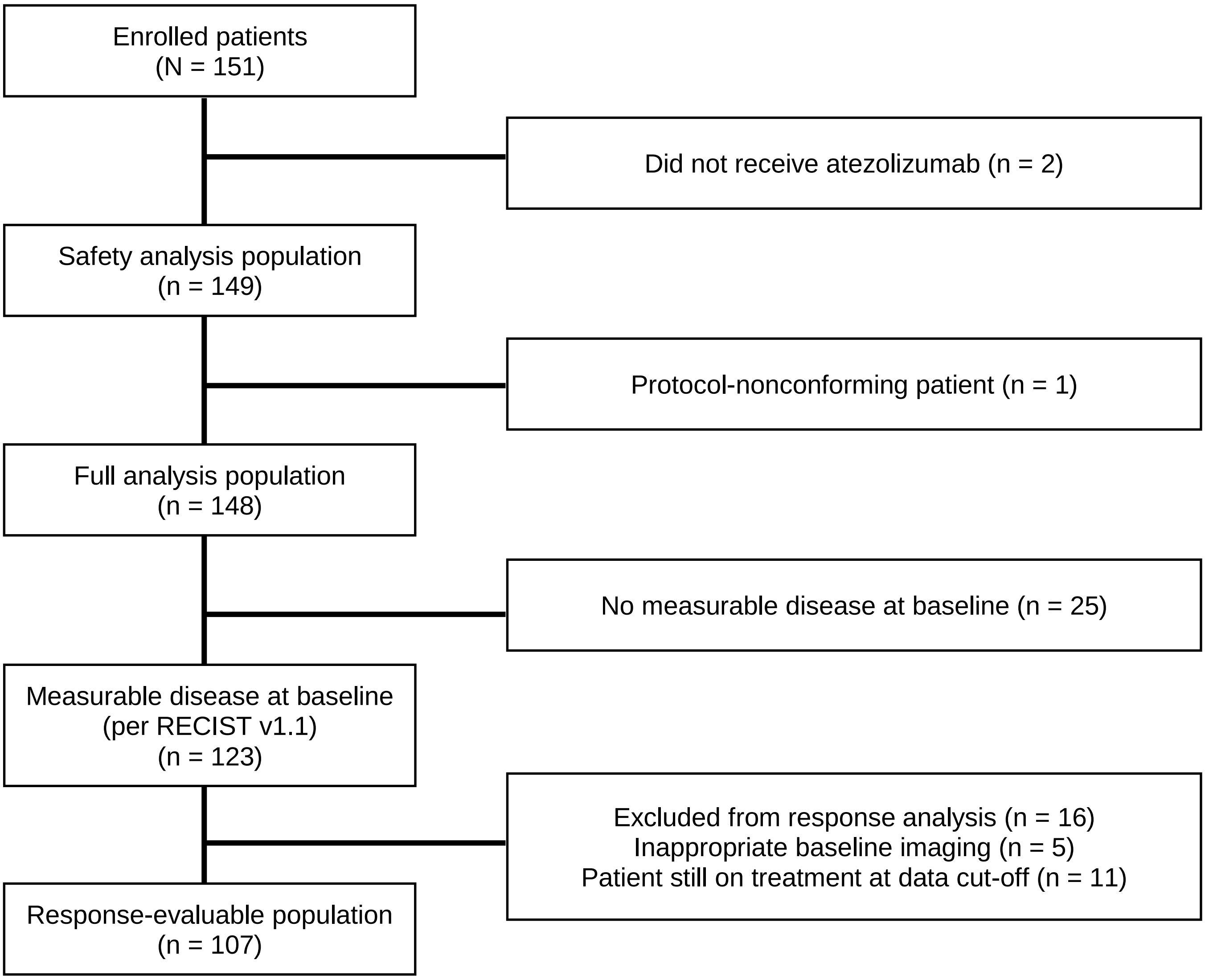

RECIST v1.1, Response Evaluation Criteria in Solid Tumours, version 1.1.

Supplementary Fig. 2. OS (full analysis population).

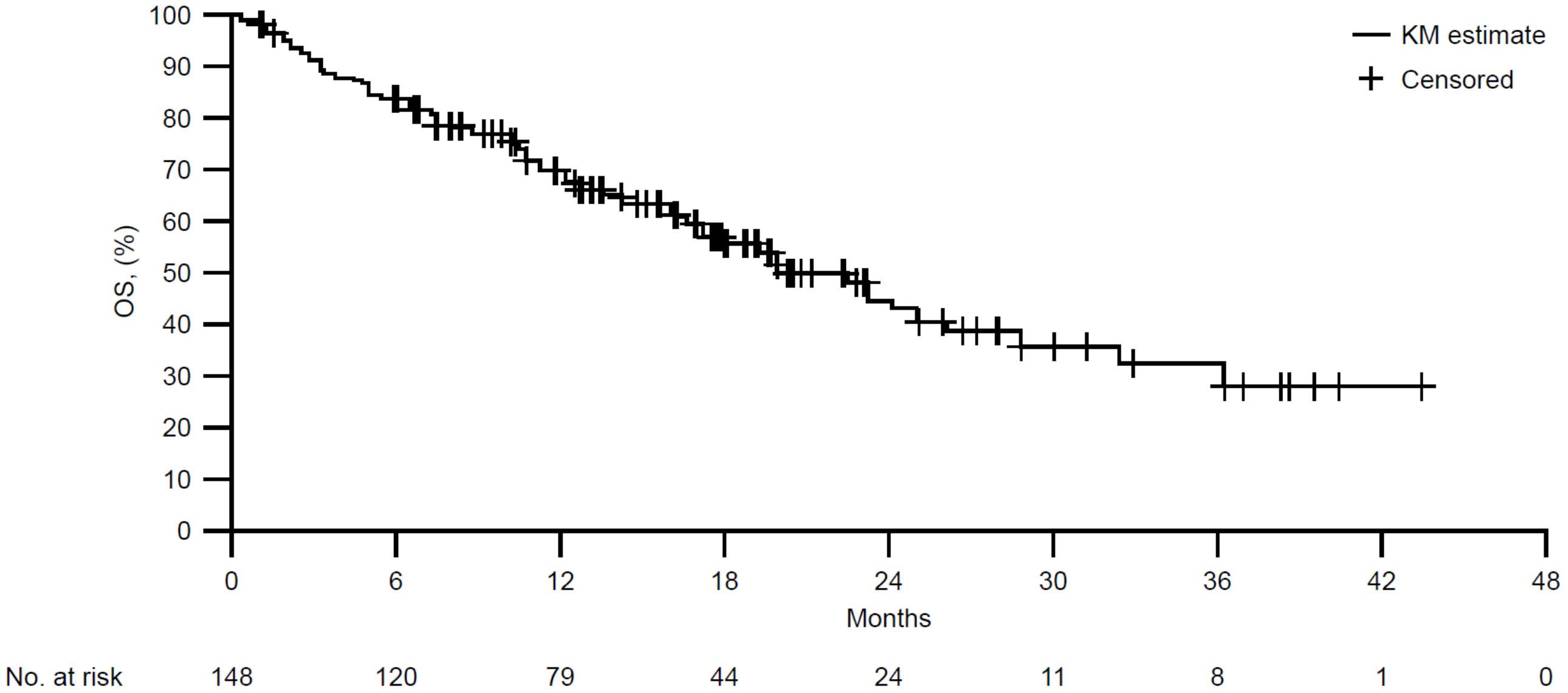

KM Kaplan–Meier, *OS* overall survival.
